# Scientific Items

**Published:** 1886-04-20

**Authors:** 


### SCIENTIFIC ITEMS.

**Balata.**—(Pharm. Jour. and Trans.) Balata is the concrete milky juice of Mimsops globosa (*Gaertner*), a large, hardwooded forest tree, sometimes reaching 120 feet in height, and found ranging from Jamaica and Trindad to Venezula and French Guiana. Balata has never found its way into commerce in any considerable quantity, though it has always been highly spoken of. It is intermediate in character between India-rubber and gutta-percha, combining the properties of both, and for certain purposes is better adapted than any other of the natural caoutchouc substances. Its strength, also, is very great, and, as it does not stretch under tension, for special appliances, such as bands for machinery, it is unequalled.

**Sight for the Blind.**—(Berlin Med. Zentral-Zeit.) The Academy of Sciences is at present engaged in testing a new and highly important discovery by Dr. Emil Martin, Fellow of the Medical Faculty of Marseilles, which is no less than restoration of sight to the blind by mechanical means. Dr. Martin's device is an apparatus of platinum so arranged as to take the place of the normal media through which light is brought into the *camera obscura* of the eye. As the painless application of the apparatus, in a neat surgical way, has already been accomplished, it is believed that a favorable outcome may be reasonably predicted for the invention.

**Galvanized Corpses.**—The Chemist and Druggist says: "Cremation and inhumation are not the only resources of civilization in regard to corpses. M. Kergovatz, chemical engineer of Guiparas (Finisterre), has advocated in the Paris Figaro a galvanoplastic treatment which he declares he has proved to be practical. He rubs the body with plumbago, and then plunges it in a bath of copper, zinc, silver, or gold, according to the funds, available, and he thus obtains a statue which cannot fail to be an accurate likeness, and which, he thinks, would be in all respects superior to the ordinary representations of great men in our streets. Besides, the actual person of the dead is retained. He declares his intention of submitting his invention to the Municipal Council of Paris." Burying, he says, has been condemned by experience; cremation destroys all evidence of crime in cases of murder, and embalming is expensive.

**Our Disappearing Forests.**—(Chicago News) The reckless improvidence with which the forests of this country are being destroyed is likely to bring its own punishment with it. Prof. Sargent's report upon the present condition of our forests has been published as a part of the tenth census. From this it appears the loss from forest fires alone amounts to upwards of $20,000,000 annually. The railways destroy upwards of 30,000,000 fine young trees every year for ties. The frame houses, which form so characteristic a feature of American landscape, use up the lumber from millions of acres annually. As yet no process of reparation has begun. We cut down, but we do not replant. The consequence is seen in the greater frequency of droughts and floods along our large rivers, the impaired fertility of the denuded soil, and the increased cost of fuel and building material. The report shows that it is quite time to turn over a new leaf. We should husband carefully what forest we have left. It is a part of wisdom for every owner of a timber tract to cut understandingly and with reference to the future. A good twenty-acre timber lot can be made to last for centuries, all the time furnishing its annual cutting of fuel for the owner.

**The Fuel Problem.**—Tests made by Dr. Fischer, the well-known German chemist, show that in the ordinary domestic stoves in use not more than 20 per cent. of the fuel consumed is really utilized for warming the rooms; whereas, with stoves burning gas, 80 per cent. and more of the possible effect is obtained. In a certain sugar manufactory at Elsdorf, it is stated, no steam engines have been used for several years. Gas is made at a cost of about twenty cents per thousand feet, and is used for lighting and for driving gas engines. At the great Schultz Iron Works, at Essen, water gas is made at a cost of about eight to sixteen cents a thousand feet, and serves both for fires and lighting. For the latter purpose a ring is fixed over the burners, having rods or pencils of magnesia attached, these being made glowing hot by the non-luminous gas flame, and emitting an excellent light. The abandonment, of burning coal direct for heating will do away with all the disadvantages of smoke.—*Ex.*

**Hearing of Infants.**—In Dr. Lindsay's "Mind in the Lower Animals," it is stated that all infants are deaf at birth because the outer ear is as yet closed, and there is no air in the middle ear. A response to a strong sound is observed, at the earliest, in six hours, but often, not for a day or two. The awakening of the sense may be observed by the irregular muscular movements and blinking which a loud noise occasions. No other organ contributes so much as the ear to the intellectual development of the child. This is shown by the intellectual backwardness of those born deaf compared with those born blind. The sense of hearing becomes early developed, so that the child soon distinguishes the different tones of those about him.

Light is at first unpleasant, and the infant shuts his eyes when brought to it. Brightness and darkness can alone be distinguished. The motions of the eyes are wholly unregulated. There is no real symmetry of movement before the first six days. The first perceptions are those of light. The child turns his head to the window within the first week. It is three weeks, however, before the eyes will follow a light that is moved before it.

Danbury makes one-fourth of all the hats worn in the United States. It turns out hourly, on an average, 1,343 hats.

